# Disrupted single-subject gray matter networks are associated with cognitive decline and cortical atrophy in Alzheimer’s disease

**DOI:** 10.3389/fnins.2024.1366761

**Published:** 2024-05-10

**Authors:** Yaqiong Xiao, Lei Gao, Yubin Hu, The Alzheimer’s Disease Neuroimaging Initiative

**Affiliations:** ^1^Center for Language and Brain, Shenzhen Institute of Neuroscience, Shenzhen, China; ^2^Department of Radiology, Zhongnan Hospital of Wuhan University, Wuhan, China

**Keywords:** Alzheimer’s disease, single-subject gray matter network, graph theory, cognitive decline, cortical atrophy

## Abstract

**Background:**

Research has shown disrupted structural network measures related to cognitive decline and future cortical atrophy during the progression of Alzheimer’s disease (AD). However, evidence regarding the individual variability of gray matter network measures and the associations with concurrent cognitive decline and cortical atrophy related to AD is still sparse.

**Objective:**

To investigate whether alterations in single-subject gray matter networks are related to concurrent cognitive decline and cortical gray matter atrophy during AD progression.

**Methods:**

We analyzed structural MRI data from 185 cognitively normal (CN), 150 mild cognitive impairment (MCI), and 153 AD participants, and calculated the global network metrics of gray matter networks for each participant. We examined the alterations of single-subject gray matter networks in patients with MCI and AD, and investigated the associations of network metrics with concurrent cognitive decline and cortical gray matter atrophy.

**Results:**

The small-world properties including gamma, lambda, and sigma had lower values in the MCI and AD groups than the CN group. AD patients had reduced degree, clustering coefficient, and path length than the CN and MCI groups. We observed significant associations of cognitive ability with degree in the CN group, with gamma and sigma in the MCI group, and with degree, connectivity density, clustering coefficient, and path length in the AD group. There were significant correlation patterns between sigma values and cortical gray matter volume in the CN, MCI, and AD groups.

**Conclusion:**

These findings suggest the individual variability of gray matter network metrics may be valuable to track concurrent cognitive decline and cortical atrophy during AD progression. This may contribute to a better understanding of cognitive decline and brain morphological alterations related to AD.

## Introduction

Alzheimer’s disease (AD) is a progressive, neurodegenerative disorder characterized by both cognitive decline and brain atrophy. Evidence from previous neuroimaging studies has shown AD-related brain morphometric changes using structural magnetic resonance imaging (MRI). There is also growing evidence to suggest disruptions of structural brain networks that are constructed using brain morphological features such as thickness and volume of (GM) in both mild cognitive impairment (MCI) and AD (He et al., 2008; Yao et al., 2010; Reid and Evans, 2013; Dai and He, 2014; Dai et al., 2019). These studies, using the graph theoretical approach, reported altered local and global network properties in MCI and AD patients, such as a greater network segregation and reduced small-world coefficient, as compared to cognitively normal (CN) subjects (He et al., 2008; Yao et al., 2010).

While network properties based on group-level structural covariance across subjects have been proven useful to reveal brain structural topological organizations, it cannot derive individual-level network measures and thus does not allow to examine the potential clinical relevance for individuals. To overcome this limitation, single-subject morphometric network analyses were developed to evaluate brain structural networks at the individual level (for reviews, see Cai et al., 2023; Wang and He, 2024). Studies have shown these analyses are robust and reliable (Sebenius et al., 2023; Yin et al., 2023), and particularly suitable for identifying biomarkers for brain disorders such as AD (Tijms et al., 2012, 2013b; Li et al., 2017; Xue et al., 2023). Findings from previous research have also demonstrated the biological relevance of the morphometric network analyses (Sun et al., 2022; Li et al., 2023). So far, several approaches have been developed to construct the single-subject morphometric networks, and different approaches estimate interregional morphological connectivity from different perspectives (for reviews, see Cai et al., 2023; Wang and He, 2024). Among the methodologies, the approach developed by Tijms et al. (2012) is widely used to estimate the interregional relationships of morphological features (i.e., GM volume) (e.g., Tijms et al., 2014, 2018; Dicks et al., 2018; Verfaillie et al., 2018; Vermunt et al., 2020). The single-subject morphological networks are further analyzed with graph theoretical approach to reveal the network’s topological properties such as degree, clustering coefficient, and small-worldness (Tijms et al., 2014; Verfaillie et al., 2018; Pelkmans et al., 2022; Wang and He, 2024).

In recent years, single-subject morphological network analyses have been widely used in the field of AD research to gain a better understanding of the brain morphometric alterations related to AD (Tijms et al., 2013b; Dicks et al., 2018; Pelkmans et al., 2022; Wang and He, 2024). For example, research has consistently reported that disrupted single-subject structural network properties are closely related to cognitive decline in both MCI and AD patients (Pereira et al., 2016; Dicks et al., 2018; Tijms et al., 2018; Verfaillie et al., 2018). Research also found different network topological patterns in distinct AD subtypes which had varying cognitive decline (Ferreira et al., 2019). In addition, GM network properties have been suggested as sensitive markers to identify the individual variability of clinical decline in patients with prodromal AD (Pelkmans et al., 2022).

More importantly, single-subject GM networks can be used to examine the associations between structural network alterations and individual differences in clinical progression in AD. Previous studies have reported that worse disruptions of network measures are related to more severe cognitive decline in AD (Tijms et al., 2013a, 2014) and predementia AD patients (Dicks et al., 2018; Tijms et al., 2018; Dicks et al., 2020; Vermunt et al., 2020; Ng et al., 2022). A few studies have demonstrated that disrupted GM networks are already present in preclinical AD patients who had normal cognition but with aggregating amyloid (Tijms et al., 2016; ten Kate et al., 2018; Voevodskaya et al., 2018; Dicks et al., 2020). Together, these studies suggest that single-subject network analysis is a sensible and feasible approach to examine individual differences within the group, and that GM network measures may serve as useful predictors of cognitive decline in the progression of AD pathology, even at the preclinical stage.

In recent studies, single-subject structural network measures have been examined across AD continuum including the CN, MCI, and AD groups (Dicks et al., 2020; Pelkmans et al., 2021; Sheng et al., 2021; Ng et al., 2022). There were greater network abnormalities in patients with more severe disease severity (Sheng et al., 2021; Ng et al., 2022), and the associations of disrupted network measures with cognitive decline depended on stages for different cognitive domains (Dicks et al., 2020). Furthermore, research has shown that structural network measures can predict future cortical atrophy in hippocampus, which is specific for preclinical AD patients but not for cognitively unimpaired normal older subjects (Dicks et al., 2020). These findings imply that single-subject network measures are useful to track and predict both cognitive decline and cortical atrophy related to disease progression in AD. However, so far, evidence regarding the associations of GM network measures with the concurrent cognitive decline and cortical gray matter atrophy during the course of AD progression is still sparse.

In this study, we aimed to investigate the alterations in topological patterns during the progression of AD and the associations of structural network measures with the concurrent cognitive decline and cortical atrophy. We constructed the single-subject morphological networks using the approach described in Tijms et al. (2012) as this approach demonstrates great reliability and stability (Tijms et al., 2012) and is widely used in the field of AD research (e.g., Tijms et al., 2014, 2018; Dicks et al., 2018; Verfaillie et al., 2018; Vermunt et al., 2020). We then calculated the global network measures of single-subject gray matter networks in CN, MCI, and AD participants. We anticipated that network measures would be altered in patients with increasing severity of AD. Next, we examined the associations of global network measures with cognitive ability and expected to observe reduced network measures associated with cognitive decline regardless of disease severity. Further, we examined the relationship between the small-world coefficient and GM volume in the CN, MCI, and AD groups. The small-world coefficient (i.e., sigma), reflecting a balance between information segmentation and integration within the network (Humphries and Gurney, 2008), is considered to be a summary of multiple topological features (i.e., normalized clustering coefficient and normalized path length). In fact, the small-world coefficient is also one of the network measures that is most robustly associated with cognitive decline (Dicks et al., 2018; Tijms et al., 2018; Verfaillie et al., 2018). We thus used this metric as the index to examine the association between GM network measures and cortical GM volume, hypothesizing that there would be different correlation patterns in CN, MCI, and AD participants at varying stages of cognitive decline.

## Materials and methods

### Participants

In this study, we used the MRI and clinical data obtained from the Alzheimer’s Disease Neuroimaging Initiative (ADNI) database.^1^ The ADNI was launched in 2003 as a public-private partnership, led by Principal Investigator Michael W. Weiner, MD. The primary goal of ADNI has been to test whether serial MRI, positron emission tomography (PET), other biological markers, and clinical and neuropsychological assessment can be combined to measure the progression of MCI and early AD. For more detailed information about this database, please refer to the website^2^ and publications (Weiner et al., 2010, 2017; Aisen et al., 2015).

Specifically, we included a total of 488 participants in the analysis: 185 CN (90 M/95 F, mean age = 74.8 ± 7.17 years, 58.4–94.7 years), 150 MCI (85 M/65 F, mean age = 73.45 ± 8.7 years, 55.2–97.4 years), and 153 AD (88 M/65F, mean age = 75.47 ± 7.74 years, 56–91 years). We selected this cohort of participants because they had quality MRI and clinical data, and these participants were matched on age and gender across groups. All the participants provided demographic information (i.e., age, gender, years of education) and clinical data, including scores from the Clinical Dementia Rating (CDR) and the Mini-Mental State Examination (MMSE). In addition, MoCA scores were available for the majority of participants (CN: *n* = 172; MCI: *n* = 100; AD: *n* = 92).

### MRI data collection

The T1-weighted structural MRI images of all 488 participants were analyzed in this study. For detailed information regarding ADNI’s image acquisition protocols which are different for multiple MRI scanner types used in ADNI, see this link.^3^ Raw Digital Imaging and Communications in Medicine (DICOM) MRI scans were downloaded from the public ADNI site.^4^ All the raw images were automatically corrected for spatial distortion caused by gradient nonlinearity and B1 field inhomogeneity.

### Structural MRI data preprocessing

Briefly, MR images were visually inspected for quality to ensure that the MRI data were usable, and then realigned to the standard anterior commissure (AC)- posterior commissure (PC) orientations for better segmentations. The preprocessing was conducted with the voxel-based morphometry (VBM) pipeline using the Computational Anatomy Toolbox (CAT12)^5^ for Statistical Parametric Mapping (SPM12),^6^ running in Matlab R2020a (MathWorks, Natick, MA, United States). All MRI data were segmented into GM, white matter, and cerebrospinal fluid images using the default tissue probability maps. After finishing segmentation, the quality of segmented images was visually checked, and all the GM images were qualified for constructing networks. The total intracranial volume (TIV) was calculated for each participant, which was used to control for different brain sizes in the statistical analyses.

### Single-subject GM network construction

Gray matter networks were constructed using a previously published, automated pipeline (Tijms et al., 2012)^7^ implemented in Matlab R2020a. The details of this pipeline has been described elsewhere (Tijms et al., 2012). Briefly, each GM segmentation was divided into 3 * 3 * 3 voxel cubes, corresponding to 6 mm * 6 mm * 6 mm for each cube. These non-overlapping cubes were served as nodes in the network and used to construct single-subject GM networks. Connections between each pair of cubes for each individual scan were established by calculating the Pearson’s correlation coefficient of the GM volume between the corresponding voxels. This approach takes into account both the gray matter probability (i.e., from the tissue segmentation) and the spatial information present in 27 voxels within each cube. The similarity matrices were binarized to construct unweighted and undirected graphs with a threshold of 5%, determined using the false discovery rate (FDR) correction for each network. To calculate small world metrics, we generated 20 random matrices by rearranging the edges of the network while keeping the spatial degree distribution intact for each binarized similarity matrix (Tijms et al., 2012, 2013a).

### Calculation of global network metrics

Next, we calculated the global network metrics for each individual GM network. Specifically, we computed the following metrics to assess the global network properties: (1) network size, the total number of nodes in the network; (2) degree, average of all the degrees (degree: number of connections that a node has) of the nodes in the network; (3) connectivity density, the percent of existing connections to the number of all possible connections within the network; (4) clustering coefficient, average clustering coefficient (the number of edges between a node and its direct neighbors) over all nodes within the network; (5) path length, the average of the shortest path length (minimal number of edges from one node to another node) over all nodes within the network.

Further, we computed the global normalized clustering coefficient (gamma), normalized path length (lambda), and small world coefficient (sigma) to estimate the small world property of the networks. The gamma and lambda are calculated by dividing the average clustering coefficient and path length values by those mean values of 20 randomized networks of identical size and degree distribution (Maslov and Sneppen, 2002). For a network to contain the small world property, it is required that gamma is greater than 1 and lambda is approximately equal to 1. And the sigma, defined as the ratio of gamma and lambda, is supposed to greater than 1 for a network to have small world property (Humphries and Gurney, 2008). All the network measures were calculated based on the functions from the Brain Connectivity Toolbox (Rubinov and Sporns, 2010).^8^

### Statistical analysis

Comparisons of demographic and clinical scores among CN, MCI, and AD groups were conducted using the chi-squared test for categorical variable (sex), one-way analysis of variance (ANOVA) for continuous variables (i.e., age, education, MMSE, and MoCA), and Kruskal-Wallis ANOVA for CDR scores.

We then examined group differences in all the network metrics using the regression analysis, controlling for age, sex, education, and TIV. For network metrics showing significant differences among CN, MCI, and AD groups, we tested the differences between groups (i.e., CN vs. MCI, CN vs. AD, and MCI vs. AD) using pairwise two-sample *t* tests with the FDR correction for multiple testing.

To analyze the relationships between the cognitive ability as measured by the MoCA (i.e., MoCA total scores) and global network metrics, we used linear regression models with the MoCA total scores as the independent variable and each of the global network metrics (i.e., degree, connectivity density, clustering coefficient, path length, gamma, lambda, and sigma) as the dependent variable, controlling for age, sex, education, and TIV. The regression analyses were first conducted across the whole sample, and then tested within each diagnostic group.

These group comparisons and regression analyses were conducted in R software (version 4.1.2).

### Associations between the small world coefficient and GM volume

Further, we examined the associations between the small-world property (i.e., sigma) and whole-brain GM volume in the CN, MCI, and AD groups, separately. Specifically, we conducted the whole brain voxel-wise correlation analysis using the “y_Correlation_Image” function in DAPBI (a toolbox for Data Processing & Analysis for Brain Imaging^9^; Yan et al., 2016), with the small-world coefficient value (i.e., sigma) as a covariate of interest, and age, sex, and TIV as covariates of no interest. The resulting *r* maps were converted to *z* maps, and the Gaussian Random Field (GRF) theory was applied for multiple comparisons correction for each correlation map with the following thresholds: voxel-wise *p* < 0.001, cluster-wise *p* < 0.05, two tailed (*z* > 3.29, GRF corrected).

## Results

### Characteristics of demographic information and clinical data

As shown in Table 1, the CN, MCI, and AD groups were matched on gender and age (*p*s > 0.05), but these groups differed (*p*s < 0.001) in years of education, CDR, MMSE, and MoCA.

**Table 1:** Demographic and clinical details in the CN, MCI, and AD groups.

	CN (*n* = 185)		MCI (*n* = 150)		AD (*n* = 153)		*p*-value
	Mean ± sd	Range	Mean ± sd	Range	Mean ± sd	Range	
Sex (M/F)	90/95		85/65		88/65		0.19
Age (years)	74.8 ± 7.17	58.4–94.7	73.45 ± 8.7	55.2–97.4	75.47 ± 7.74	56–91	0.075
Education (years)	16.83 ± 2.36	12–20	15.8 ± 3	6–20	15.59 ± 2.64	8–20	<0.001
CDR	0 ± 0	0–0	0.53 ± 0.16	0.5–2	0.81 ± 0.36	0.5–2	<0.001
MMSE	29.06 ± 1.13	24–30	26.83 ± 2.54	16–30	22.89 ± 2.96	5–30	<0.001
MoCA	24.3 ± 1.95	18–29	21.83 ± 4.6	4–29	17.89 ± 5.4	0–27	<0.001

CN, cognitively normal; MCI, mild cognitive impairment; AD, Alzheimer’s disease; CDR, Clinical Dementia Rating; MMSE, Mini Mental State Examination; MoCA, Montreal Cognitive Assessment.

### Single-subject GM networks and group differences in network metrics

**Figure 1:**
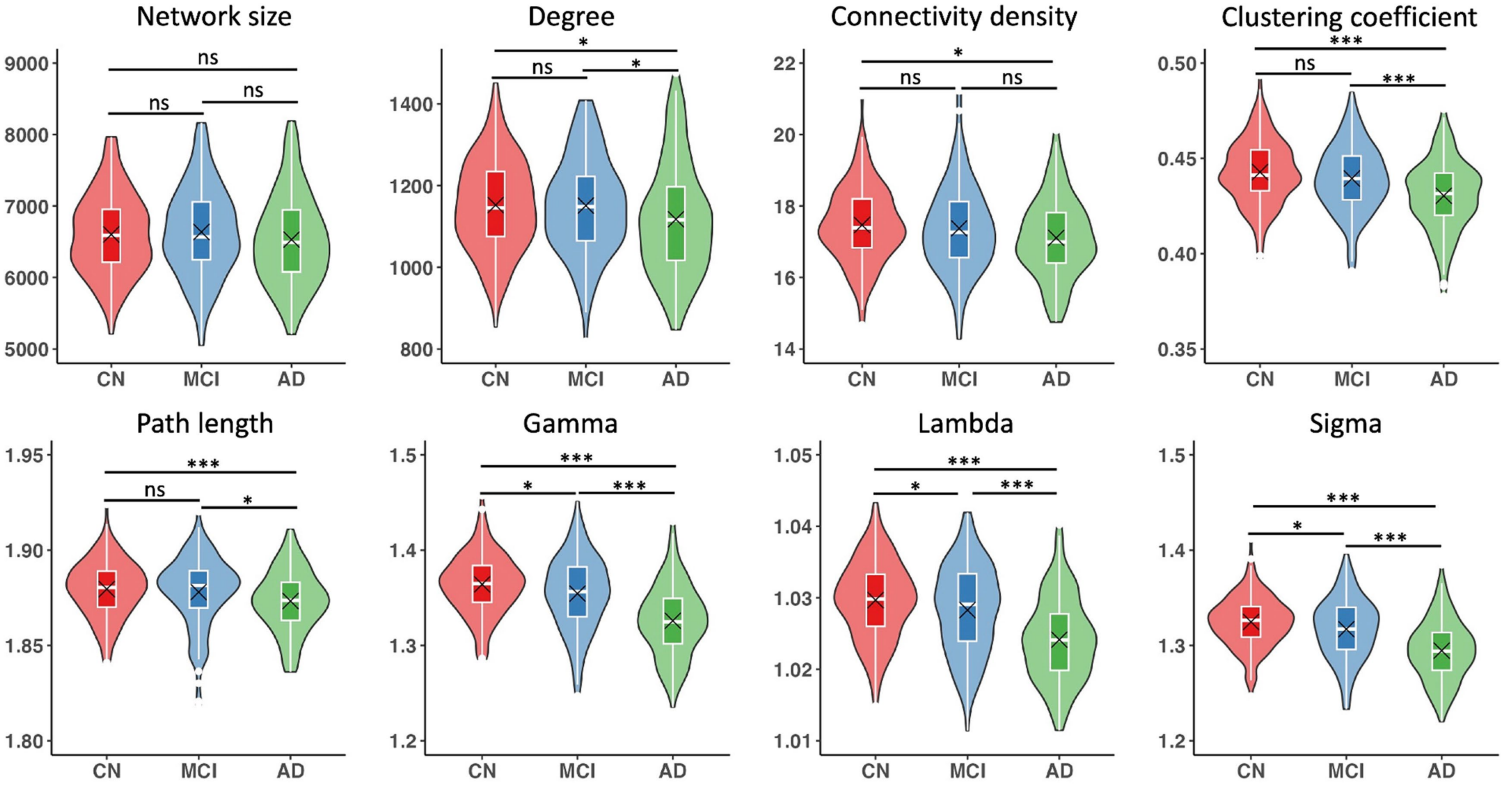
Single-subject gray matter network metrics and comparisons between CN, MCI, and AD groups. Boxplots show interquartile range (IQR; first quartile, Q1; third quartile, Q3); the horizontal line inside the box represents the median. Black cross indicates the mean value. Significant results were corrected for multiple comparisons using the FDR method. *p < 0.05, ***p < 0.001, ns, not significant.

Networks had an average size of 6,590 nodes (sd = 608) and an average connectivity density of 17.3% (sd = 1.1) across all participants. Although all single-subject gray matter networks in the CN, MCI, and AD groups had a small world topology (i.e., sigma >1), the topology was altered in both MCI and AD groups as compared to the CN group (CN vs. MCI: *p* = 0.02; CN vs. AD: *p* < 0.001), controlling for age, sex, education, and TIV (Table 2). We observed that almost all the network metrics showed lower values in the MCI or AD group than the CN group (Table 2; Figure 1). Specifically, compared to the CN group, both MCI and AD patients had lower gamma (CN vs. MCI: *p* = 0.01; CN vs. AD: *p* < 0.001), lambda (CN vs. MCI: *p* = 0.03; CN vs. AD: *p* < 0.001), and sigma (CN vs. MCI: *p* = 0.02; CN vs. AD: *p* < 0.001). In addition, AD patients had reduced degree (*p* = 0.03), connectivity density (*p* = 0.01), clustering coefficient (*p* < 0.001), and path length (*p* = 0.002) than the CN group. As compared to MCI patients, AD patients had reduced degree (*p* = 0.03), clustering coefficient (*p* < 0.001), path length (*p* = 0.02), gamma (*p* < 0.001), lambda (*p* < 0.001), and sigma (*p* < 0.001). All the results were corrected for multiple comparisons using the FDR method.

**Table 2:** Summary of global network metrics and group differences between CN, MCI, and AD groups.

	CN (*n* = 185)	MCI (*n* = 150)	AD (*n* = 153)	*p*-value^a^	*p* value^b^	*p*-value^c^	*p*-value^d^
Network size	6602.12 ± 564.01	6636.67 ± 630.29	6529.56 ± 634.6	0.01	0.6	0.41	0.38
Degree	1152.34 ± 112.4	1150.94 ± 117.2	1117.44 ± 136.22	0.0007	0.92	0.03	0.03
Connectivity density	17.47 ± 1.05	17.38 ± 1.23	17.11 ± 1.1	0.009	0.45	0.01	0.06
Clustering coefficient	0.443 ± 0.02	0.439 ± 0.02	0.43 ± 0.02	<0.001	0.08	<0.001	<0.001
Path length	1.88 ± 0.01	1.878 ± 0.02	1.87 ± 0.02	0.002	0.4	0.002	0.02
Gamma	1.36 ± 0.03	1.35 ± 0.04	1.33 ± 0.04	<0.001	0.01	<0.001	<0.001
Lambda	1.03 ± 0.005	1.029 ± 0.006	1.024 ± 0.006	<0.001	0.03	<0.001	<0.001
Sigma	1.325 ± 0.03	1.317 ± 0.03	1.295 ± 0.03	<0.001	0.02	<0.001	<0.001

Data are presented as mean ± sd; CN, cognitively normal; MCI, mild cognitive impairment; AD, Alzheimer’s disease. ^a^Statistical results from comparisons among the CN, MCI, and MCI groups, controlling for age, sex, education, and TIV. ^b^Statistical results from comparisons between the CN and MCI groups. ^c^Statistical results from comparisons between the CN and AD groups. ^d^Statistical results from comparisons between the MCI and AD groups.

### Relationships between network metrics and cognitive ability

**Figure 2:**
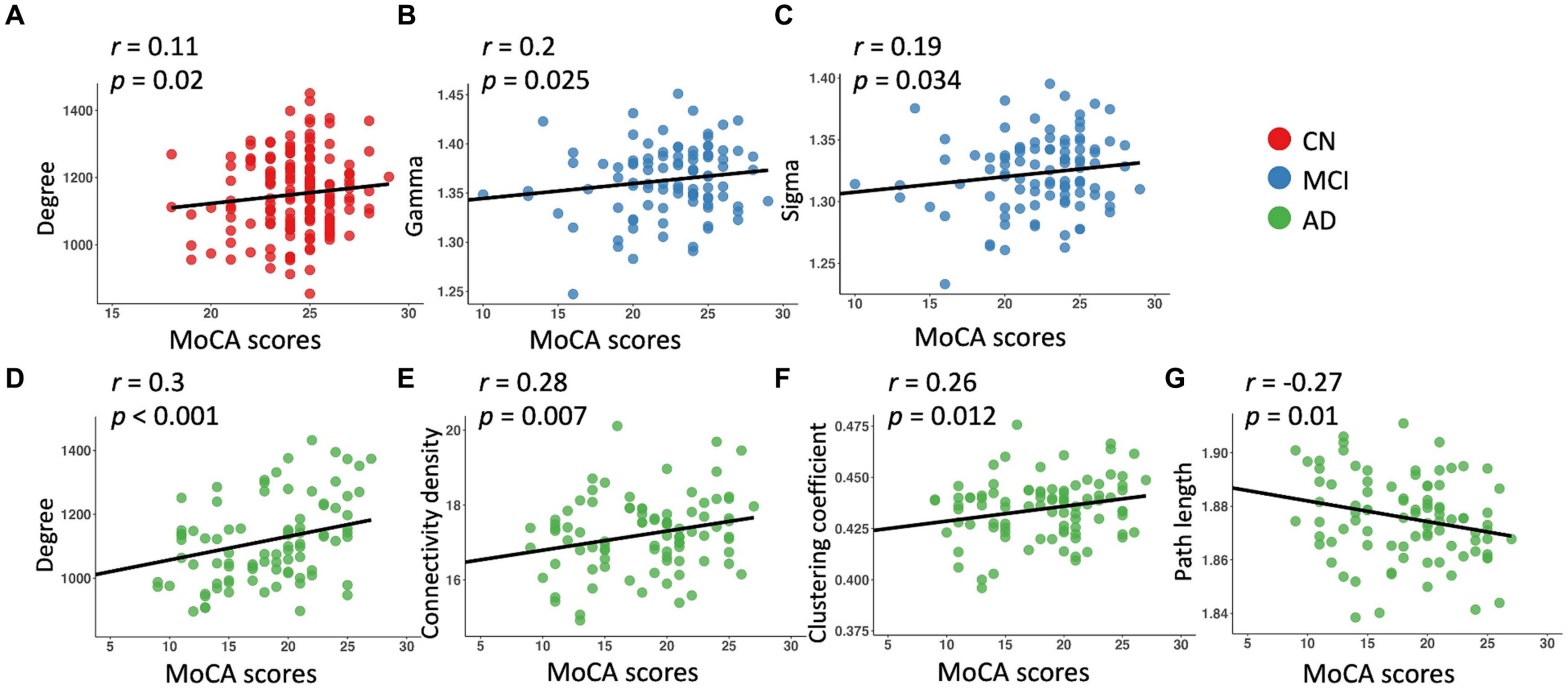
Linear regression model fitted plots between MoCA scores and network metrics in the CN **(A)**, MCI **(B,C)**, and AD **(D–G)** groups. The *r*-values were from the Pearson’s correlation analysis, and *p* values were from the regression analyses, controlling for age, sex, education, and TIV.

Across the whole sample, significant relationships with MoCA scores were found in all the network metrics except path length, controlling for age, sex, education, and TIV (Table 3). We observed significant associations of MoCA scores with degree (*β* = 5.22, *p* = 0.2) in the CN group, and significant associations of MoCA scores with gamma (*β* = 0.002, *p* = 0.025) and sigma (*β* = 0.001, *p* = 0.034) in the MCI group (Table 3; Figure 2). In the AD group, MoCA scores were significantly correlated with network metrics including degree (*β* = 6.6, *p* < 0.001), connectivity density (*β* = 0.05, *p* = 0.007), clustering coefficient (*β* = 0.0008, *p* = 0.012), and path length (*β* = −0.0007, *p* = 0.01) (Table 3; Figure 2).

**Table 3:** Correlations between gray matter network metrics and MoCA scores, controlling for age, sex, education, and TIV.

	Whole sample (*n* = 488)	CN (*n* = 185)	MCI (*n* = 150)	AD (*n* = 153)
Degree	**< 0.001**	**0.02**	0.382	**< 0.001**
Connectivity density	**0.024**	0.208	0.234	**0.007**
Clustering coefficient	**< 0.001**	0.323	0.821	**0.012**
Path length	0.85	0.228	0.081	**0.01**
Gamma	**<0.001**	0.323	**0.025**	0.253
Lambda	**0.002**	0.6	0.076	0.171
Sigma	**<0.001**	0.213	**0.034**	0.116

CN, cognitively normal; MCI, mild cognitive impairment; AD, Alzheimer’s disease. Significant correlation coefficients (i.e., *p*-values) are highlighted in bold.

### Significant associations between the small-world coefficient and cortical GM volume

**Figure 3:**
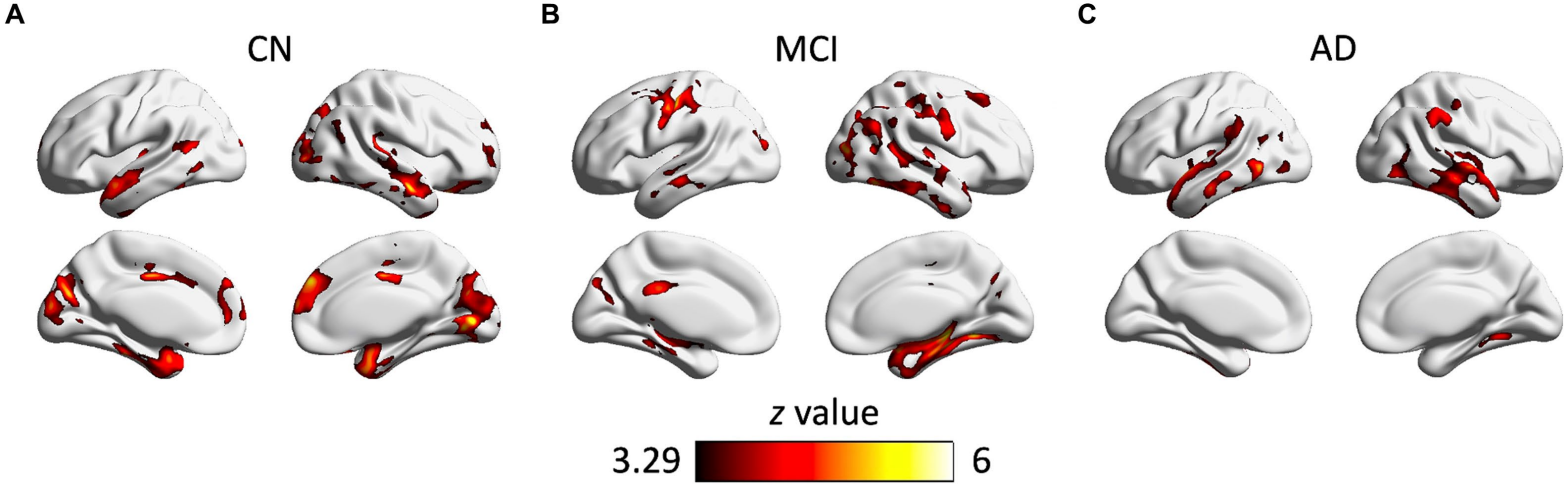
Clusters showing significant associations between the small-world coefficient and cortical gray matter volume in the CN **(A)**, MCI **(B)**, and AD **(C)** groups, controlling for age, gender, and TIV. The resulting clusters were corrected for multiple comparisons (*z* > 3.29, GRF corrected). CN, cognitively normal; MCI, mild cognitive impairment; AD, Alzheimer’s disease.

As shown in Figure 3, there were significant associations between the small-world coefficient and cortical GM volume in the CN, MCI, and AD groups, controlling for age, sex, and TIV. Specifically, significant correlations were observed in brain regions including bilateral middle temporal gyrus, superior temporal gyrus, inferior temporal gyrus, parahippocampal gyrus, fusiform gyrus, inferior parietal lobe, insula, middle occipital gyrus, inferior occipital gyrus, and lingual gyrus (Table 4). Additional significant clusters in amygdala, Heschl’s gyrus, cuneus, precuneus, posterior cingulate cortex (PCC), calcarine, middle cingulate cortex (MCC), superior occipital gyrus, and right cerebellum posterior lobe were observed in the CN group. There were also additional significant clusters found in amygdala, postcentral gyrus, angular gyrus, middle frontal gyrus, precuneus, cuneus, middle occipital gyrus, calcarine, MCC, and PCC in the MCI group (Table 4). All the resulting clusters were corrected for multiple comparisons using the GRF method with the following thresholds: voxel-wise *p* < 0.001, cluster size >1,300 voxels, cluster-wise *p* < 0.05, two tailed (*Z* > 3.29).

**Table 4:** Brain regions showing significant associations between the small-world coefficient (sigma) and gray matter volume in the CN, MCI, and AD groups, separately.

Region	Cluster size (voxels)	Peak MNI coordinates			Peak *Z* value
		*x*	*y*	*z*	
CN group					
Right STG, MTG, ITG, SFG, parahippocampal gyrus, IFG, insula, fusiform gyrus, amygdala, IPL	23,182	21	5	−41	5.68
Left MTG, STG, ITG, parahippocampal gyrus, insula, amygdala, Heschl’s gyrus	11,710	−44	2	−27	5.52
Left cuneus, precuneus, PCC, calcarine, lingual gyrus, SOG	16,705	−12	32	30	4.8
Bilateral MCC	2,819	−5	−12	41	5.27
Left MTG, STG, ITG	2,299	−62	−53	11	5.64
Right cerebellum posterior lobe	1840	15	−74	−38	4.98
MCI group					
Right ITG, STG, MTG, IPL, parahippocampal gyrus, MOG, postcentral gyrus, hippocampus, precuneus, insula, angular gyrus, lingual gyrus, IFG	36,349	51	−54	−18	6
Left MTG, parahippocampal gyrus, hippocampus, STG, insula, ITG, fusiform gyrus, amygdala, IFG	8,249	−36	−23	−6	4.58
Left postcentral gyrus, MFG, IPL	7,974	−54	−15	48	5.52
Left precuneus, cuneus, MOG, calcarine, SOG	3,375	−21	−80	24	4.4
Bilateral MCC, PCC	1,447	−6	−32	30	4.8
AD group					
Left MTG, STG, ITG, fusiform gyrus, MOG, IPL, parahippocampal gyrus, supramarginal gyrus, insula, IOG	18,918	−65	−8	−6	5.68
Right MTG, STG, ITG, fusiform gyrus, IPL, insula, MOG, lingual gyrus, IOG, parahippocampal gyrus	16,534	63	−11	−6	5.12

MTG, middle temporal gyrus; STG, superior temporal gyrus; ITG, inferior temporal gyrus; IPL, inferior parietal lobe; SFG, superior frontal gyrus; IFG, inferior frontal gyrus; MFG, middle frontal gyrus; SOG, superior occipital gyrus; MOG, middle occipital gyrus; IOG, inferior occipital gyrus; MCC, middle cingulate cortex; PCC, posterior cingulate cortex.

## Discussion

In the present study, we observed AD-related alterations in the structural network topological patterns by comparisons of single-subject GM network measures. We also demonstrated significant relationships between disrupted global network metrics and cognitive decline for both the whole sample and each diagnostic group. Importantly, we observed significant associations of the small-world coefficient with cortical GM volume in brain regions including temporal cortex, parahippocampal gyrus, occipital cortex, parietal cortex, fusiform gyrus, and insula in the CN, MCI, and AD groups, with additional regions involved in the CN and MCI groups. These findings provide further evidence for the altered structural network properties related to the disease severity of AD and suggest the individual variability of GM network metrics may be valuable and sensible markers for the concurrent cognitive decline and brain morphologic alterations during the progression of AD.

One of the main findings is that all three groups showed significant associations between the small-world coefficient and the concurrent GM volume in a variety of brain regions. The prominent clusters included bilateral temporal cortex, parahippocampal gyrus, occipital cortex, and parietal cortex, which have been recognized as vulnerable brain regions to show atrophy in both normal aging (Glodzik et al., 2012) and AD progression (Hampel et al., 2008; Shi et al., 2009; Frisoni et al., 2010; Albert et al., 2011). This finding suggests the reduced small-world coefficient in individuals is related to the concurrent GM atrophy in these vulnerable regions. Notably, the frontal cortex, cuneus, MCC, PCC, and amygdala were additionally found in the CN and MCI groups, suggesting thta the small-world coefficient might capture more morphological alterations in normal aging adults and patients at early stages of dementia. Our finding here extends previous reports that network measures predict future hippocampal atrophy for preclinical AD subjects (Dicks et al., 2020), and demonstrates that the reduced small-world coefficient is also related to GM volume atrophy in normal aging adults and patients at different stages of dementia.

One possible biological basis of the associations between the small-world coefficient and GM volume is that alterations in GM volume may lead to rewiring of neural circuits, affecting the efficiency of information transmission and integration across the brain network (Crossley et al., 2014; Xiong et al., 2023). Another possible biological basis is that disruptions in the network organization, as reflected by the network measures (e.g., small-world coefficient), may impact GM volume through mechanisms such as neuronal degeneration, synaptic pruning, and synaptic plasticity (Meftah and Gan, 2023). It is also possible that there may be shared genetic, molecular, or cellular mechanisms underlying GM volume changes and alterations in morphological networks (Pol et al., 2006). Further investigations utilizing functional measures like functional MRI or anatomical measures such as diffusion tensor imaging are still needed to elucidate the intricate neurobiological underpinnings of these associations observed here.

In AD patients, network measures including degree, connectivity density, clustering coefficient, and path length were associated with concurrent cognitive ability. This finding is in line with previous studies that reported reduced network measures related to cognitive dysfunctions in AD (Tijms et al., 2013a, 2014; Vermunt et al., 2020). The associations of network measures and cognitive ability were also found in the CN and MCI groups. Specifically, we observed significant associations of degree with cognitive ability in CN individuals, indicating that reduced global degree is associated with cognitive decline in normal aging. Previous cross-sectional and longitudinal studies in CN participants have consistently reported that brain networks tend to reorganize toward a more random topology (i.e., low clustering coefficient, low path length) with age (Chen et al., 2011; Zhu et al., 2012; Wu et al., 2013; Carey et al., 2019). However, we did not observe such correlations in the CN group. Instead, we found correlations of small-world properties (including gamma and sigma) with the cognitive ability in the MCI group. These results might indicate that disrupted network measures reflect cognitive decline as seen in normal aging adults and patients with dementia, especially in AD patients. Importantly, the associations between network measures and the concurrent cognitive function in cognitively unimpaired normal older individuals and patients at early and later stages of dementia also support the idea that network measures may provide a biological substrate for cognitive dysfunction during normal aging and AD progression (Pereira et al., 2016; Pelkmans et al., 2021, 2022).

We observed that small-world properties (i.e., gamma >1, lambda ~1, sigma >1) exist in all groups, indicating that all participants including MCI and AD patients still exhibited a relatively higher level of network integration and better local communication as compared to the characteristics of random networks. Nevertheless, single-subject GM networks in both the MCI and AD groups were characterized by a more random topology than the CN group, as indicated by a decreased clustering coefficient, path length, and small-world properties (including gamma, lambda, and sigma values). The topological patterns were much more random in AD patients than MCI patients, reflecting the disruptions of GM network were related to the disease severity in AD. These findings are in line with previous structural (Tijms et al., 2013a; Sheng et al., 2021; Ng et al., 2022) and functional (Stam et al., 2009; Sanz-Arigita et al., 2010) network studies in AD.

The present study has two main limitations that are worth noting. First, neither preclinical AD nor asymptomatic control participants who later developed dementia were included here. In future studies, these participants should also be included to gain a complete picture of alterations in brain structural networks during the pathological progression of AD. Second, this study lacked longitudinal data, which prevented the examination of network disruption trajectories concurrent with cognitive decline or GM atrophy. Thus, longitudinal studies are still needed to identify early network markers for the brain morphological changes related to preclinical dementia and AD. Additionally, the findings reported here may be biased by the individual variability of the cross-sectional samples.

## Conclusion

Our study provides new evidence for brain morphological alterations related to AD using a graph theoretical approach to capture the characteristics of single-subject GM networks. Altered network metrics were correlated with the concurrent cognitive decline in normal aging adults and patients with dementia (including MCI and AD), suggesting that the brain structure topological organization may predict the severity of the cognitive decline, especially in AD patients. Further, we presented associations between the small-world coefficient (i.e., sigma) and cortical GM volume in the temporal cortex, parahippocampal gyrus, occipital cortex, parietal cortex, fusiform gyrus, and insula in the CN, MCI, and AD groups. These brain regions are vulnerable to atrophy during normal aging and AD progression. Our findings suggest that single-subject GM network metrics may be sensible markers for detecting the concurrent cognitive decline and cortical GM atrophy, highlighting the potential for applying structural network metrics to track the disease progression of AD.

## Data availability statement

The datasets presented in this study can be found in online repositories. The names of the repository/repositories and accession number(s) can be found at: the Alzheimer’s Disease Neuroimaging Initiative (ADNI) database (adni.loni.usc.edu) and the following link: https://github.com/Yaqiongxiao/individual.GM.network.AD.

## Ethics statement

The Ethics committees/institutional review boards that approved the ADNI study are: Albany Medical Center Committee on Research Involving Human Subjects Institutional Review Board, Boston University Medical Campus and Boston Medical Center Institutional Review Board, Butler Hospital Institutional Review Board, Cleveland Clinic Institutional Review Board, Columbia University Medical Center Institutional Review Board, Duke University Health System Institutional Review Board, Emory Institutional Review Board, Georgetown University Institutional Review Board, Health Sciences Institutional Review Board, Houston Methodist Institutional Review Board, Howard University Office of Regulatory Research Compliance, Icahn School of Medicine at Mount Sinai Program for the Protection of Human Subjects, Indiana University Institutional Review Board, Institutional Review Board of Baylor College of Medicine, Jewish General Hospital Research Ethics Board, Johns Hopkins Medicine Institutional Review Board, Lifespan - Rhode Island Hospital Institutional Review Board, Mayo Clinic Institutional Review Board, Mount Sinai Medical Center Institutional Review Board, Nathan Kline Institute for Psychiatric Research & Rockland Psychiatric Center Institutional Review Board, New York University Langone Medical Center School of Medicine Institutional Review Board, Northwestern University Institutional Review Board, Oregon Health and Science University Institutional Review Board, Partners Human Research Committee Research Ethics, Board Sunnybrook Health Sciences Centre, Roper St. Francis Healthcare Institutional Review Board, Rush University Medical Center Institutional Review Board, St. Joseph’s Phoenix Institutional Review Board, Stanford Institutional Review Board, The Ohio State University Institutional Review Board, University Hospitals Cleveland Medical Center Institutional Review Board, University of Alabama Office of the IRB, University of British Columbia Research Ethics Board, University of California Davis Institutional Review Board Administration, University of California Los Angeles Office of the Human Research Protection Program, University of California San Diego Human Research Protections Program, University of California San Francisco Human Research Protection Program, University of Iowa Institutional Review Board, University of Kansas Medical Center Human Subjects Committee, University of Kentucky Medical Institutional Review Board, University of Michigan Medical School Institutional Review Board, University of Pennsylvania Institutional Review Board, University of Pittsburgh Institutional Review Board, University of Rochester Research Subjects Review Board, University of South Florida Institutional Review Board, University of Southern, California Institutional Review Board, UT Southwestern Institution Review Board, VA Long Beach Healthcare System Institutional Review Board, Vanderbilt University Medical Center Institutional Review Board, Wake Forest School of Medicine Institutional Review Board, Washington University School of Medicine Institutional Review Board, Western Institutional Review Board, Western University Health Sciences Research Ethics Board, and Yale University Institutional Review Board. The studies were conducted in accordance with the local legislation and institutional requirements. The participants provided their written informed consent to participate in this study. Written informed consent was obtained from the individual(s) for the publication of any potentially identifiable images or data included in this article.

## Author contributions

YX: Conceptualization, Data curation, Formal analysis, Funding acquisition, Writing – original draft, Writing – review & editing. LG: Conceptualization, Writing – review & editing. YH: Data curation, Validation, Writing – review & editing.

## Alzheimer’s Disease Neuroimaging Initiative

Data used in preparation of this article were obtained from the Alzheimer’s Disease Neuroimaging Initiative (ADNI) database (adni.loni.usc.edu). As such, the investigators within the ADNI contributed to the design and implementation of ADNI and/or provided data but did not participate in analysis or writing of this report. A complete listing of ADNI investigators can be found at: http://adni.loni.usc.edu/wp-content/uploads/how_to_apply/ADNI_Acknowledgement_List.pdf.

## Footnotes

^1^ https://adni.loni.usc.edu/

^2^ http://www.adni-info.org

^3^ http://adni.loni.usc.edu/methods/documents/mri-protocols/

^4^ http://www.loni.ucla.edu/ADNI

^5^ https://neuro-jena.github.io/cat/

^6^ https://www.fil.ion.ucl.ac.uk/spm/software/spm12/

^7^ https://github.com/bettytijms/Single_Subject_Grey_Matter_Networks

^8^ https://sites.google.com/site/bctnet/

^9^ https://rfmri.org/dpabi
